# Ceramic-on-Ceramic Bearing in Total Hip Arthroplasty Reduces the Risk for Revision for Periprosthetic Joint Infection Compared to Ceramic-on-Polyethylene: A Matched Analysis of 118,753 Cementless THA Based on the German Arthroplasty Registry

**DOI:** 10.3390/jcm10061193

**Published:** 2021-03-12

**Authors:** Lisa Renner, Carsten Perka, Oliver Melsheimer, Alexander Grimberg, Volkmar Jansson, Arnd Steinbrück

**Affiliations:** 1 Department of Orthopaedics, Center for Musculoskeletal Surgery, Charité—University Medicine, Charitéplatz 1, 10117 Berlin, Germany; Carsten.perka@charite.de; 2 German Arthroplasty Registry (EPRD Deutsche Endoprothesenregister gGmbH), Straße des 17. Juni 106-108, 10623 Berlin, Germany; melsheimer@eprd.de (O.M.); grimberg@eprd.de (A.G.); volkmar.jansson@med.uni-muenchen.de (V.J.); arnd.steinbrueck@med.uni-muenchen.de (A.S.); 3 Department of Orthopaedic Surgery, Physical Medicine and Rehabilitation, University Hospital of Munich (LMU), Marchioninistr. 15, 81377 Munich, Germany

**Keywords:** periprosthetic joint infection, primary total hip, bearing, ceramic-on-ceramic

## Abstract

Periprosthetic joint infection (PJI) is one of the most common complications in total hip arthroplasty (THA). The influence of bearing material on the risk of PJI remains unclear to date. This registry-based matched study investigates the role of bearing partners in primary cementless THA. Primary cementless THAs recorded in the German Arthroplasty Registry since 2012 with either a ceramic-on-ceramic (CoC) or ceramic-on-polyethylene (CoP) bearings were included in the analysis. Using propensity score matching (PSM) for age, sex, obesity, diabetes mellitus, Elixhauser comorbidity index, year of surgery and head size, we compared the risk for revision for PJI for CoC and CoP. Within the 115,538 THAs (87.1% CoP; 12.9% CoC), 977 revisions were performed due to PJI. There was a significantly higher risk for revision for PJI for CoP compared with CoC over the whole study period (*p* < 0.01) after 2:1 matching (CoP:CoC) with a hazard ratio of 1.41 (95% confidence interval (CI), 1.09 to 1.80) After 3 years, the risk for revision for PJI was 0.7% (CI 0.5–0.9%) for CoC and 0.9% (CI 0.8–1.1%) for CoP. The risk for revision for all other reasons except PJI did not significantly differ between the two groups over the whole study period (*p* = 0.4). Cementless THAs with CoC bearings were less likely to be revised because of infection in mid-term follow-up. In the future, registry-embedded studies focusing on long-term follow-up, including clinical data, as well as basic science studies, may give a deeper insight into the influence of the bearing partners.

## 1. Introduction

Periprosthetic joint infection (PJI) is one of the most common reasons for revision in primary total hip arthroplasty (THA) and causes devastating complications for patients as well as high costs for health systems [1,2]. Multiple risk factors for PJI have been identified, whereby diabetes and obesity may be the most important patient-related modifiable ones [3,4]. The rate of PJI after THA for patients with primary osteoarthritis, however, has stayed between 1 and 3% over the last 30 years [5,6,7], so research is increasingly focusing on innovative aspects such as the actual prosthesis, the fixation method or the material of bearing partners. Metal-on-metal (MoM) bearings are said to be associated with a higher risk of infection [8,9]. This may be due to a change in periprosthetic tissue or an altered immunologic response because of metallic debris, thus leading surgeons to abandon stemmed MoM THA [10]. The other hard-on-hard bearing, ceramic-on-ceramic (CoC), is said to outperform metal-on-polyethylene (MoP) and ceramic-on-polyethylene (CoP) regarding wear and longevity, and has been often used, especially in young patients, since the 1990s [11,12]. However mechanical problems such as squeaking, breakage, incorrect ceramic insertion or impingement postoperatively display the technical expertise regarding surgical technique [13]. Low wear is less likely to cause synovitis, effusion and hyperemia, and ceramic particles are more biocompatible than metal debris [14], raising the question of the influence of CoC on infection. However, there are limited data on CoC reducing the rate of PJI, as recent studies investigating this topic may not have precisely captured important covariables [15,16]. A current meta-analysis including 11 randomized controlled studies and six observational studies did not demonstrate a significant difference in risk of PJI in relation to bearing combination in THA [17]. Studies based on registry data are an appropriate methodology to further analyze this connection, as registries have been shown to be superior for detecting early trends. The German Arthroplasty Registry uses an elaborate system to capture almost all revision surgeries regardless of which hospital in Germany they were performed in [18,19].

The current study investigates the risk of PJI in relation to the bearing partner in primary cementless THA based on data from the German Arthroplasty Registry. We hypothesized that CoC bearings would have a lower risk for revision for PJI compared with CoP.

## 2. Materials and Methods

### 2.1. Data Collection

The German Arthroplasty Registry (Endoprothesenregister Deutschland, EPRD) started collecting data in November 2012 and works as a not-for-profit organization founded by surgeons and the German Society of Orthopedics and Orthopedic Surgery (DGOOC) in cooperation with public health insurers (AOK Bundesverband GbR, Verband der Ersatzkassen e.V vdek), the German Medical Technology Association (BVMed), and hospitals performing hip and knee arthroplasty. Despite reporting surgeries being voluntarily, approximately 70% of primary and revision total hip and knee arthroplasties were reported in 2019 [1]. The two participating insurance companies (AOK-B, vdek) cover around 65% of the German population and allocate important information given by hospitals to cross-validate. A revision of a THA registered in the EPRD will be followed up due to the insurance billing date, even if it is performed in a hospital which is not participating in the German Arthoplasty Registry. Except for procedures performed outside of Germany, this algorithm ensures a close to perfect follow-up of patients insured by these companies [18]. Within the EPRD, the German versions of the International Classification of Procedures in Medicine (ICPM), called the “Operationen und Prozedurenschlüssel” (OPS) 301 system, and of the 10th International Classification of Diseases (ICD-10) are used to classify and identify diagnosis and procedures accurately.

### 2.2. Study Subjects

We included all patients with primary osteoarthritis of the hip and dysplastic hip osteoarthritis receiving a cementless THA with either a ceramic-on-ceramic or a ceramic-on-polyethylene bearing. Exclusion criteria were patients with secondary osteoarthritis and post-traumatic osteoarthritis of the hip, patients with relevant previous surgeries and patients receiving a cemented or hybrid THA. Two groups were formed based on the bearing combination: CoP and CoC. The primary endpoint in our study was revision for PJI. The secondary endpoint was defined as revision for any reason other than PJI. The EPRD uses a 2-step approach to safely identify PJI. First, while entering information for each case into the EPRD database, the surgeon is asked to classify the reason for revision. In a second step, if PJI is flagged, this is directly reported to the EPRD via electronic case report form (eCRF) or when reimbursement data are coded as ICD-10 T84.5. This information is linked within the EPRD database.

### 2.3. Statistical Analysis

We originally considered using a multivariate Cox regression for assessing the effects of the bearing type and the potential confounders. However, as the proportional hazard assumption for the Cox regression was rejected, we switched to propensity score matching (PSM) for elimination of confounders and only examined the influence of the bearing type. The covariables considered for PSM were age, sex, obesity, diabetes mellitus complication, Elixhauser comorbidity index, head size and year of surgery. Elixhauser comorbidity index, obesity and diabetes complication were determined using ICD-10-codes from insurance company data based on the coding algorithm defined by Quan et al. [20]. R was used to conduct the statistical analysis [21]. For the statistical analysis after matching, Kaplan–Meier estimates were calculated, log-rank tests were performed and the hazard ratios computed for the matched data. To assess covariate balance after matching, standardized mean differences (SMD) were used.

## 3. Results

**Figure 1:**
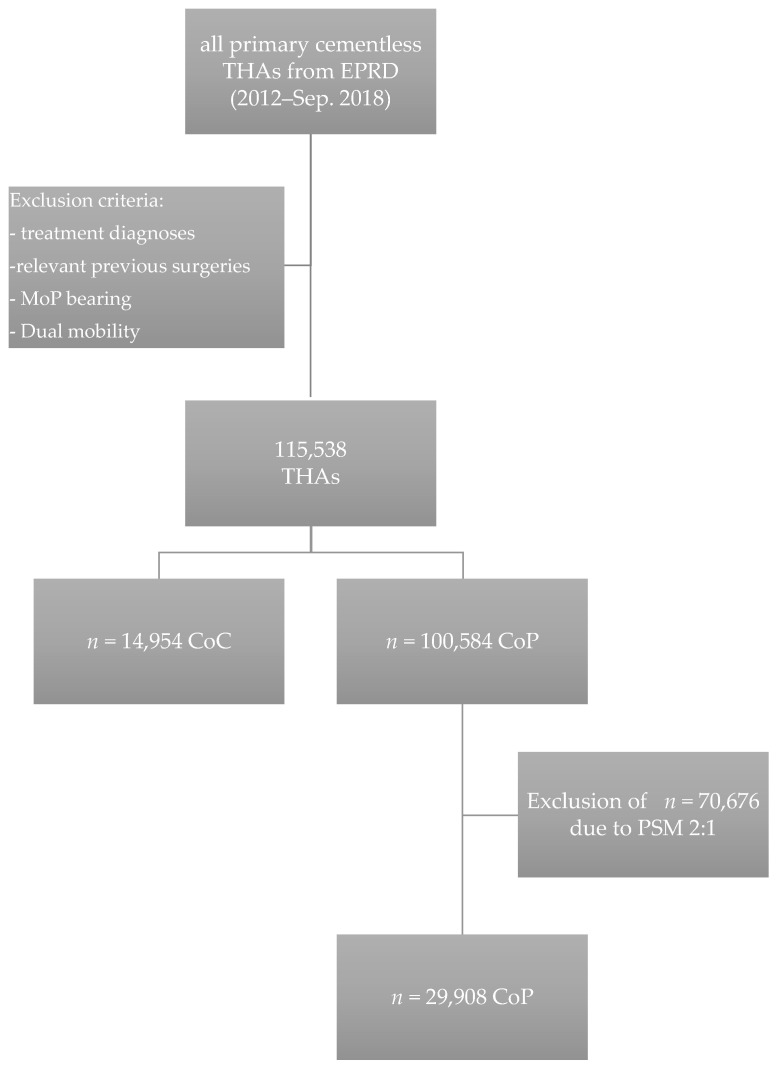
Flowchart illustrating the data acquisition within the German Arthroplasty Registry (EPRD) as well as 1:2 matching using the propensity score (THA, total hip arthroplasty; EPRD, German Arthroplasty Registry; MoP Metal-on-Polyethylen; CoC, Ceramic-on -Ceramic; CoP, Ceramic-on-Polyethylene; PSM, Propensity Score Matching).

The final dataset consisted of 115,538 cases, of which 14,954 cases had a ceramic-on-ceramic bearing (12.9%) and 100,954 (87.1%) had a ceramic-on-polyethylene or ceramic-on-crosslinked polyethylene bearing (Figure 1).

Overall, 3027 (2.6%) revisions for any reason occurred in the whole study group, of which 977 (0.8%) were performed due to PJI. There were 83 revisions due to PJI in the CoC group (0.6%) and 894 in the CoP group (0.9%) before performing PSM. The crude data show a difference in age at date of surgery, with patients being older within the CoP group (mean, 67.4 years; standard deviation (SD) 9.6) compared with the CoC group (median, 62.6 years; SD 10.2), as well as differences in gender distribution and other important cofactors like head size (Table 1).

**Figure 2:**
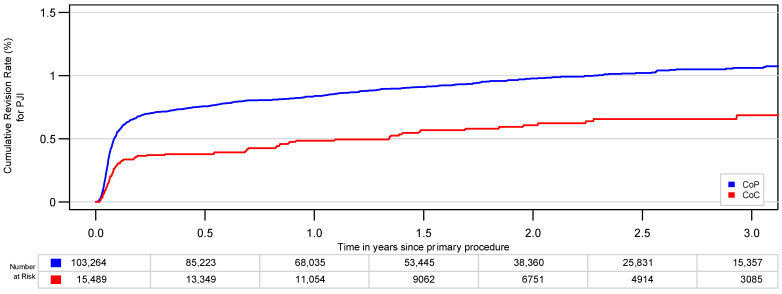
Cumulative risk of revision for periprosthetic joint infection (PJI) of ceramic-on-polyethylene (CoP) and ceramic-on-ceramic (CoC) bearings before matching (*p* < 0.01).

The crude risk of revision (CRR) for PJI was 0.7% (95% confidence interval [CI], 0.5–0.9%) for CoC and 1.1% (CI, 1.0–1.1%) for CoP after 3 years (Figure 2). There was a statistically significant difference over the whole study period (*p* < 0.01).

**Figure 3:**
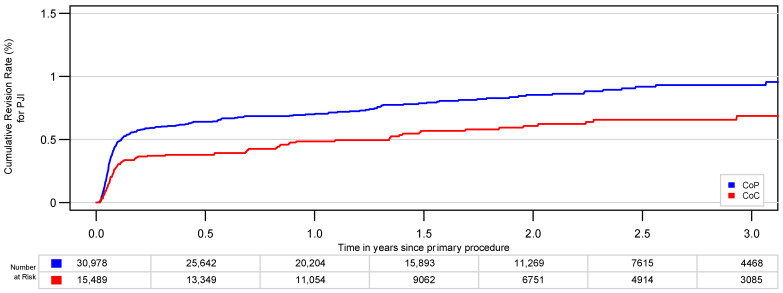
The cumulative risk of revision CoP and CoC bearings for PJI after PSM (2:1) is significantly different (*p* = 0.008).

After PSM with a 1:2 matching, 14,954 (CoC) and 29,908 cases (CoP) were included for further analysis (Figure 1, Table 1); 1:3 matching was not possible due to remaining differences in age and comorbidities. After matching, the risk for revision for PJI was 0.7% (95% CI, 0.5–0.9%) for CoC and 0.9% (95% CI, 0.8–1.1%) for CoP after 3 years (Table 2). There was a significant higher risk for infection for CoP compared with CoC using the log-rank test over the whole study period (*p* = 0.008) (Figure 3). CoP THAs were at higher risk for revision for PJI, with a hazard ratio (HR) of 1.41 (95% CI, 1.09–1.81)

**Figure 4:**
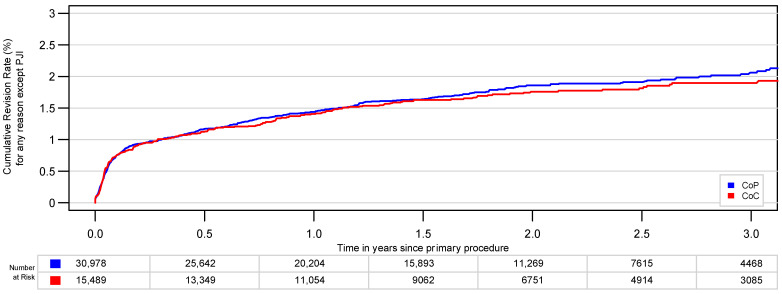
The cumulative risk of revision for any reason without PJI for CoP and CoC bearings after PSM (2:1) is not significantly different (*p* = 0.4).

There was no statistically significant difference in risk for revision for any reason except PJI (*p* = 0.4), with a CRR of 1.9% (95% CI, 1.6–2.2%) for CoC and a CRR of 2.0% (95% CI, 1.8–2.2%) for CoP after 3 years (Figure 4). The hazard ratio for CoP was 1.07 (95% CI, 0.91 to 1.2).

## 4. Discussion

This registry-based study investigated the effect of bearing partners in primary THA on the risk of revision for PJI. The most important finding of our study is a significantly lower risk for PJI in THA with a CoC compared with those with a CoP bearing. The influence of significant covariables was minimized in our analysis; more precisely, age, sex, obesity, diabetes mellitus complication, Elixhauser comorbidity index, head size and year of surgery. Furthermore, the risk for any revision except PJI did not differ significantly between the two bearings, which strengthens our hypothesis.

First, detection of the true incidence of PJI by itself is a major challenge and is especially important for generating valid data from a registry. According to the literature, registries tend to underrate PJI in total hip and knee arthroplasty [22]. In most registries, diagnosis of PJI is established before or directly after surgery; however, in some cases, microbiological results become positive days after surgery, when the diagnosis has already been reported to the database of the registry or is not changed subsequently. Within the current study, the risk of revision for PJI was around 0.8%, which is consistent with scientific data [6,7]. The EPRD ensures this in a two-step approach. First, while entering cases into the EPRD database manually, surgeons are asked to classify the reason for revision. Secondly, cases with PJI are identified using the coding based on the German versions of the International Classification of Procedures in Medicine (ICPM). Even if surgeons may have wrongly assessed the reason for revision before surgery, hospital settlements based on all of the information available at discharge of patients may include the right diagnoses.

Other registry studies investigating the question of the influence of bearing partners on the risk of PJI demonstrated that there is a time-dependent effect, with differences occurring only after 6 months [15], or an age-dependent influence, with the advantages of CoC only seen for patients younger than 70 years [16]. However, the rate of revision for PJI seems to be quite low within these studies, being 0.5% over 15 years [15,16]. Lenguerrand et al. [23] published a prospective observational study investigating a large number of factors influencing the risk of PJI in THA in 623,253 primary hip procedures in England and Wales. Bearing partners significantly influenced the risk of revision, with CoC and CoP having a significantly lower risk for PJI than MoP; again, the influence varied over time.

Furthermore, correct accounting for confounding factors in clinical and registry studies is a huge challenge. A current prospective multicenter study including 2107 first revisions of primary total hip arthroplasties with CoC bearings had a significantly higher revision rate due to PJI compared with MoP [13]; however, within this prospective observational analysis, no further information on the patients was provided and therefore, no multivariant analysis was conducted. The most recent metanalysis comparing MoP, CoP and CoC, including 11 randomized controlled trials and six observational studies [17], showed no significant differences between the bearing combinations; furthermore, the results displayed a trend towards CoP being the one with the least number of revision due to PJI. The study further suggested “adequately controlled registry studies”, as, due to their nature, registry studies represent the best way to pinpoint a previously unspecified phenomenon. The EPRD has access to various covariables and comorbidities. Propensity score matching was used to exclude the influence of the most important covariates (such as the Elixhauser comorbidity index [24], obesity, and diabetes [3]) that predict whether a patient will receive a particular treatment; in our case, the bearing combination.

Basic science suggests that despite careful preparation and sterile perioperative conditions, there may be bacterial contamination on surgical draping and attachment of bacteria at the area of operation. The fact that contamination develops into infection may be influenced by the susceptibility of the bearing combination [25], whereby the production of a biofilm, a complex glycocalyx, is the crucial step for establishing an infection by protecting against the immune system and also antibiotics. The characteristics of the surface, such as roughness and hydrophobicity, can influence the attachment of different bacteria [26,27]. Ceramics in arthroplasty are known to have a low surface roughness and high hydrophilicity and wettability, resulting in uniformly distributed synovial fluid between the bearing partners This reduces friction and wear, ensuring excellent survival in the long-term follow-up, but may also provide lower bacterial attachment. *Staphylococcus aureus*, as one of the main bacteria causing PJI, is more prone to adhering to hydrophobic surfaces than hydrophilic surfaces [28]. Lass et al. [29] detected the highest bacterial load on polyethylene liners in a retrieval study. Evidently, due to the nature of these studies, as well as our study, we can only assume an association between a lower risk for PJI and ceramic-on-ceramic bearing in THA, not causality.

Our study has the following limitations: (1) the EPRD only started collecting cases in 2012, so our follow-up is limited. However, as our aim was to focus on early-onset PJI, the follow-up time is sufficient to answer our research question. (2) Due to the limited number of cases, we were not able to divide between conventional and highly crosslinked polyethylene. Furthermore, metal heads were not included in our research protocol, as these are only used in significantly older patients in Germany. Nevertheless, our main focus was to investigate the difference between CoP and CoC bearings. (3) The EPRD lacks some covariates that might have skewed the results in risk for PJI (e.g., ASA (American Society of Anesthesiologists) classification). Furthermore, center or surgeon were not included in our analysis. (4) As this was a registry-based study, we were not able to include histologic and microbiologic intraoperative findings, nor we were able to fully execute a commonly used diagnostic tool for defining PJI like the MSIS (Musculoskeletal Infection Society) criteria [30]. Furthermore, we cannot depict the site of infection as we did not perform a retrieval analysis of the components. Still, a registry study may represent an opportunity to approach this specific phenomenon. Future clinical randomized trials or registry-embedded studies are needed to establish causality.

## 5. Conclusions

CoC bearings were less likely to be revised for PJI in our registry-based study compared with THAs with a CoP bearing. There was no difference in risk for revision for any reason except PJI. However, studies including a long-term follow-up as well as microbiologic and histologic data are needed to further support our results in the future.

## Figures and Tables

**Table 1:** Description of categorical variables stratified by bearing type before and after 1:2 matching, with standardized mean differences (SMD) indicating balance of the matched groups.

		Unmatched	Matched 2:1				SMD
Variable	Characteristics	CoP	CoP		CoC		
mean age at surgery	years (SD)	67.4 (9.8)	62.7 (10.1)		62.6 (10.2)		0.012
		%	*n*	%	*n*	%	
sex	male	39.1	12,491	41.8	6406	42.8	0.022
	female	60.9	17,417	58.2	8548	57.22	
Complicated diabetes	no	98.8	29,638	99.1	14,779	98.8	0.026
	yes	1.2	270	0.9	175	1.2	
obesity	no	81.3	24,712	82.6	12,374	82.7	0.003
	yes	18.7	5196	17.4	2580	17.3	
head size category	<32 mm	6.0	713	2.4	371	2.5	0.010
	32 mm	59.5	11,245	37.6	5557	37.2	
	>32 mm	34.5	17,950	60.0	9026	60.4	
Elixhauser index	≥5	3.0	770	2.6	410	2.7	0.014
	0	22.8	8499	28.4	4304	28.8	
	1–4	74.1	20,639	69.0	10,240	68.5	
year of surgery	2012–2014 2015 2016 2017 2018	6.3 14.1 25.3 30.1 24.1	2575 5155 7447 8436 6295	8.6 17.2 24.9 28.2 21.0	1401 2627 3741 4150 3035	9.4 17.6 25.0 27.8 20.3	0.033

**Table 2:** Cumulative probability of revision (CPR) after propensity score matching (PSM) at 1:2 for PJI comparing CoC and CoP.

	CPR (95% CI) and Numbers at Risk since Primary THA					
	1 year		2 years		3 years	
CoC	0.5 (0.4; 0.6)	10,625	0.6 (0.4; 0.6)	6485	0.7 (0.5; 0.9)	2971
CoP	0.7 (0.6; 0.8)	20,694	0.8 (0.7; 1.0)	12,588	0.9 (0.8; 1.1)	5714

## Data Availability

Data available on request due to restrictions. The data presented in this study are available on request from the corresponding author. The data are not publicly available due to data protection.
